# Identification of Genes Uniquely Expressed in the Germ-Line Tissues of the Jewel Wasp *Nasonia vitripennis*

**DOI:** 10.1534/g3.115.021386

**Published:** 2015-10-09

**Authors:** Patrick M. Ferree, Christopher Fang, Mariah Mastrodimos, Bruce A. Hay, Henry Amrhein, Omar S. Akbari

**Affiliations:** * W. M. Keck Science Department of Claremont McKenna, Pitzer, and Scripps Colleges, Claremont, California 91711; † Division of Biology and Biological Engineering (BBE), MC156-29, California Institute of Technology, Pasadena, California 91125; ‡ Department of Entomology, University of California, Riverside Center for Disease Vector Research, Institute for Integrative Genome Biology, University of California, Riverside, California 92521

**Keywords:** *Nasonia*, chromatin, ovary, testis, transcriptome

## Abstract

The jewel wasp *Nasonia vitripennis* is a rising model organism for the study of haplo-diploid reproduction characteristic of hymenopteran insects, which include all wasps, bees, and ants. We performed transcriptional profiling of the ovary, the female soma, and the male soma of *N. vitripennis* to complement a previously existing transcriptome of the wasp testis. These data were deposited into an open-access genome browser for visualization of transcripts relative to their gene models. We used these data to identify the assemblies of genes uniquely expressed in the germ-line tissues. We found that 156 protein-coding genes are expressed exclusively in the wasp testis compared with only 22 in the ovary. Of the testis-specific genes, eight are candidates for male-specific DNA packaging proteins known as protamines. We found very similar expression patterns of centrosome associated genes in the testis and ovary, arguing that *de novo* centrosome formation, a key process for development of unfertilized eggs into males, likely does not rely on large-scale transcriptional differences between these tissues. In contrast, a number of meiosis-related genes show a bias toward testis-specific expression, despite the lack of true meiosis in *N. vitripennis* males. These patterns may reflect an unexpected complexity of male gamete production in the haploid males of this organism. Broadly, these data add to the growing number of genomic and genetic tools available in *N. vitripennis* for addressing important biological questions in this rising insect model organism.

Hymenopteran insects, including all wasps, bees, and ants, are one of the most successful groups in nature; they comprise approximately 8% of all described species (Davis *et al.* 2010). Additionally, several wasp and bee species are of enormous agricultural importance because of their primary roles in the control of insect pests and pollination, respectively (Southwick and Southwick 1992; Smith 1996). An important goal for more comprehensively understanding the biology of these insects is to discern their unique mode of reproduction, haplo-diploidy, in which females arise as diploids from fertilized eggs whereas males develop as haploids from unfertilized eggs. Like in all insects, female hymenopterans store sperm in a gland called the spermatheca, which allows females to fertilize and lay eggs anywhere from days to weeks after mating (Pitnick *et al.* 1999; Collins *et al.* 2004). However, in hymenopterans not all eggs are fertilized. This “choice”—to fertilize or not—is responsible for producing both sexes and ultimately establishing the sex ratios of these insects.

Several unique cellular features are essential for hymenopteran females to produce unfertilized eggs that can develop properly into viable and fertile haploid males. One such feature regards the nature of centrosome formation. In almost all cellular contexts, new centrosomes arise solely through duplication from previous ones (Hinchcliffe and Sluder 2001). In purely diploid organisms, the sperm, in addition to contributing half of the hereditary material, provides a centrosome derived from the sperm tail basal body that is essential for organizing the first mitotic spindle of the embryo (Schatten 1994). However, in hymenopterans, unfertilized eggs destined to become males do not have access to the sperm-derived centrosome. As a solution, hymenopteran eggs can form their own centrosomes *de novo* from factors present in the egg’s cytoplasm (Callaini *et al.* 1999). Hundreds of these *de novo* centrosomes form spontaneously in the egg immediately after egg laying; in unfertilized eggs, two of these centrosomes unite with the female-derived nuclear material to facilitate its entry into the first embryonic mitosis (Tram and Sullivan 2000). However, if the egg is fertilized, the sperm-derived centrosome preferentially initiates the mitotic divisions, whereas the *de novo* centrosomes go unused and disintegrate (Tram and Sullivan 2000). Previous studies demonstrated that these *de novo* centrosomes contain γ-tubulin and CP190 (Callaini *et al.* 1999; Ferree *et al.* 2006), core components common to all centrosomes (Schatten 1994; Bettencourt-Dias and Glover 2007). However, little is known about other centrosome-associated factors that may be uniquely present in *de novo* centrosomes compared to conventional ones. A plausible scenario is that *de novo* centrosome formation relies on the expression of certain centrosome-associated genes uniquely in the female germ line so that their encoded proteins become maternally loaded in the egg cytoplasm. Alternatively, *de novo* centrosome formation may rely on posttranslational regulation of certain centrosome components specifically in the egg, or a combination of transcriptional and post-transcriptional regulation. An important step toward distinguishing among these possibilities is to identify the complete sets of centrosome-associated genes expressed in each of the hymenopteran’s germ-line tissues.

Another unique feature of haploid male development in hymenopterans pertains to sperm formation. In diploid animals, this process involves a diploid spermatocyte entering into conventional meiosis, in which homologous chromatid pairs at each chromosome position align together and then undergo two successive divisions, thereby giving rise to haploid gametes (Whiting 1968). This pattern of conventional meiosis likely occurs in hymenopteran females because they are diploid and are known to undergo normal recombination (Whiting 1968). However, in hymenopteran males, conventional meiosis cannot occur because only one chromosome set is present in this sex (Whiting 1968). Instead, sperm production in hymenopteran males may involve a modified form of mitosis that does not require homologous chromatid pairs, a hypothesis that has been supported by early cytological observations (Bull 1982). Work in traditional diploid model organisms including *Drosophila melanogaster* has led to the identification of numerous genes involved in meiosis (Sekelsky *et al.* 1999; Reis *et al.* 2011). Some intriguing questions are whether the orthologs of these genes are expressed in the male germ line of hymenopteran insects and, if so, do their gene products perform new or additional functions in this sex?

Addressing these questions is achievable by use of the jewel wasp *Nasonia vitripennis* as an experimental model organism for hymenopteran insects. A parasitoid of several blowfly species, *N. vitripennis* has been used extensively in genetic and cell biological studies to investigate a number of phenomena, including the evolution and development of axis pattern formation (Pultz *et al.* 2005; Lynch *et al.* 2006a,b), venom production (Danneels *et al.* 2010; De Graaf *et al.* 2010), sex determination (Verhulst *et al.* 2010), and host−symbiont interactions (Bordenstein *et al.* 2001; Ferree *et al.* 2008). Additionally, high-quality genomes have been produced for *N. vitripennis* and two of its sibling species, *N. giraulti* and *N. longicornis*, making possible comparative genomic studies within and among these species (Loehlin *et al.* 2010; Werren *et al.* 2010; Desjardins *et al.* 2013). Transcriptome-based investigations are further fostering *N. vitripennis* as a hymenopteran model system. One study used RNA-Seq-based transcriptional profiling to identify novel genes expressed from a “selfish” B chromosome known as paternal sex ratio, which resides natively in certain wasp populations and causes complete elimination of the paternal genome through some unknown chromatin-remodeling event(s) in the testis (Akbari *et al.* 2013). This work also produced the complete set of transcripts present in the wild type wasp testis, including those generated from a large number of new transcribed regions as well as from genes represented in the annotated reference genome (Akbari *et al.* 2013). A more recent study examining whole-genome gene expression patterns in *N. vitripennis* and *N. giraulti* revealed large-scale, sex-biased expression of genes involved in sex pheromone signaling, immunity, venom production, and cuticle synthesis in whole males and epigenetic-related genes in whole females (Wang *et al.* 2015). However, additional studies are needed to identify which specific assemblies of genes are expressed preferentially within individual tissues and developmental stages.

Here we performed transcriptional profiling of the *N. vitripennis* ovary as well as the adult male and female soma (without germ line) of this organism in order to complement a previously obtained transcriptome of the *N. vitripennis* testis (Akbari *et al.* 2013). Broadly, these data will collectively serve as an important genomic research tool for further functional and evolutionary studies; to this end, we have deposited these data into a Web-based browser for easy visualization of individual transcripts relative to their gene models (www.nasonia.caltech.edu). We used these data sets here to identify genes that are enriched and expressed exclusively in the germ-line tissues; these genes have a high likelihood of performing important and unique roles in gamete formation. For example, we report more than 150 protein-coding genes that are expressed solely in the testis. In particular, eight of these genes encode highly basic proteins that are strong candidates for protamines, rapidly evolving histone-like proteins that tightly package DNA in sperm. We also used these data to identify and examine the expression patterns of germ-line−expressed *N. vitripennis* orthologs of *D. melanogaster* genes involved in centrosome dynamics and meiosis because these genes may underlie the development of haploid males and their ability to produce gametes. With very few exceptions, the sets of centrosome-associated genes that are expressed in the ovary and testis in general do not differ, suggesting that *de novo* centrosome formation may rely primarily on post-translational and not large transcription-level differences between these tissues. Of 16 orthologs of *D. melanogaster* meiosis genes, all are expressed in the *N. vitripennis* testis, despite the lack of true meiosis in males. Interestingly, three of these genes are only expressed in the wasp testis, together suggesting functional diversification of these genes in Nasonia. Broadly, these studies highlight the effectiveness of using transcriptome data sets as tools to help address fundamental questions pertaining to hymenopteran cell and developmental biology.

## Materials and Methods

### RNA extraction, sequencing, and validation

Total RNA was extracted with the Ambion mirVana mRNA isolation kit (Ambion/Applied Biosystems, Austin, TX). Samples were then flash frozen. The male testes were extracted from 3-d-old pupae in the yellow body-red eye stage and the male carcass (lacking testes) was collected for analysis. We used the male testes sequencing data from our previous study (Akbari *et al.* 2013). The female ovaries were extracted from 3-d-old adult females and these were collected for analysis along with their corresponding carcass samples (lacking ovaries). After extraction, RNA was treated with Ambion Turbo DNase (Ambion/Applied Biosystems, Austin, TX). The quality of RNA was assessed using the Bioanalyzer 2100 (Aglient Technologies, Santa Clara, CA) and the NanoDrop 1000 UV-VIS spectrophotometer (NanoDrop Technologies/Thermo Scientific, Wilmington, DE). RNA was then prepared for sequencing using the Illumina mRNA-Seq Sample Preparation Kit (Illumina, San Diego, CA) and the Illumina HiSequation 2500 sequencer was used for sequencing.

PolyA transcriptome reads for the male testes (36,225,986 reads), male carcass (32,708,044 reads), female ovaries (42,744,507 reads), and female carcass (43,508,877 reads) were processed and aligned to a reference index generated for the *Nasonia vitripennis* genome Nvit_2.0 (obtained from www.ncbi.nlm.nih.gov) and transcriptome Nvit_OGSv1.2 (obtained from www.hymenopteragenome.org/), using TopHat v2.0.9 (Trapnell *et al.* 2009). Reads were aligned using default parameters allowing up to 40 alignments per read with a maximum 2-bp mismatch.

The amino acid composition analysis for identification of Arginine-rich genes was calculated by using Perl scripts that can be provided on request.

For reverse-transcription polymerase chain reaction (RT-PCR), tissues were dissected into 1× phosphate-buffered saline. Trizol Reagent (Ambion) was used to extract total RNA. Samples were treated with DNase and then DNase Inactivator Reagent (Ambion). Complementary DNA from each sample was synthesized by using the iScript cDNA Synthesis Kit (Bio-Rad). PCR amplification was carried out with the following primer sequences: for NV17569, Forward CTG CAC TTC TTC ATC CCT GG and Reverse GGA ATC CAC TCG ACG AAG TAC; for NV16796, Forward CAT CGG AGC CAC GAA TAG AC and Reverse AGT ACA AAG GGC TTG AAG ATC G; for NV16651, Forward TGG AGC TTG TCG TTA TTG TCG and Reverse CAT TGT GAA CAG CAA CAC GAG; for NV16652, Forward GGA CAG GCG GTA ATT CCA TC and Reverse GAA CGG AGA CAG CTT GGT ATC; for NV12055, Forward GTT CGG CAA CTG TAC TCT GTA G and Reverse GGA GTT CTT GGG TAG TAG TAT GC; and for NV10574, Forward ATG CTC TCC AAT GAC CAA CG and Reverse AGT TGT CAA GTC TCC TCC AAG. Thermocycler reactions were conducted with the following program: 92° for 2 min, 35 cycles of 92° for 30 sec, 54° for 30 sec, and 72° for 30 sec, followed by a final extension time of 5 min at 72°.

### Data availability

All raw RNA-Seq data will be submitted to the NCBI short read archive and links will be made available at www.nasonia.caltech.edu. The assembled reads are publicly available in our Nasonia genome browser located at www.nasonia.caltech.edu.

## Results

**Figure 1:**
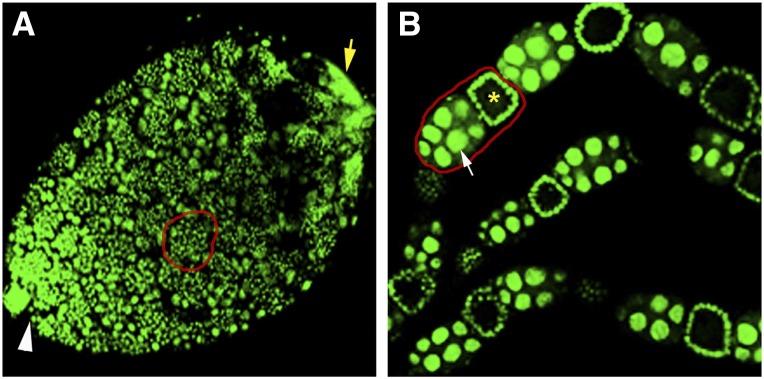
Confocal images of 4′,6-diamidino-2-phenylindole (DAPI)-stained ovaries and testes. (A) Testis of a yellow body-red eye pupa. White arrowhead marks the anterior end where stem cell division occurs, while yellow arrow marks the posterior testis end containing the elongating spermatids. Red circle highlights a cyst of dividing spermatocytes. (B) A section of three ovarioles taken from an adult female wasp. Red circle shows an egg chamber containing the oocyte (yellow asterisk) and nurse cells (white arrow). Younger oocytes and mature eggs are also present in the ovarioles but are not shown due to the length of the whole ovary.

We previously performed transcriptional profiling of testes taken from *N. vitripennis* pupae in the yellow body-red eye developmental stage (Akbari *et al.* 2013). This stage was chosen because testes at this time exhibit the widest range of spermatogenesis stages, from early dividing cystocytes through early stages of sperm individualization (Figure 1A), thus including the transcriptionally active periods. To complement this study, we performed RNA-Seq on isolated ovaries from adult females, which contain all stages of oogenesis, from germ line stem cell division to mature egg (Figure 1B). We also profiled total transcripts from the male adult carcass and female adult carcass from which the germ-line tissues were completely removed. Using these samples, we performed RNA-Seq analysis using the Illumina platform (see *Materials and Methods*), producing a total of 155,187,414 reads with 142,314,705 (91.71%) mapping to the wasp genome (Supporting Information, Table S1). This particular combination of datasets allowed us to identify (*i*) the genes expressed in each of these two germ-line and two somatic tissue conditions and (*ii*) the genes expressed uniquely in each germ-line tissue (*i.e.*, to the exclusion of the other germ-line tissue and both somatic conditions). In total, we discovered 8355 and 9891 genes of 20,813 genes present in the *N. vitripennis* reference genome that were expressed [fragments per kilobase of transcript per million (FPKM) ≥1)] in the transcriptomes of the ovary and testis, respectively (Table S2 and Table S3). Additionally, 8675 and 8575 genes were expressed in the female and male adult somatic tissues, respectively (Table S2 and Table S3). Genes including yellow-G (CG5717) and yellow-G2 (CG13804), expressed solely in the *D. melanogaster* ovary (Graveley *et al.* 2011), have *N. vitripennis* orthologs NV16651, NV16652, NV16653, and NV16654 that are also expressed exclusively in the wasp ovary. Similarly, the *N. vitripennis* genes NV17569 and NV15938, which are orthologs of the testis-specific *D. melanogaster* genes βTub85D (CG9359) and CG9222 (FBgn0031784) (Graveley *et al.* 2011), respectively, were expressed solely in the wasp testis. These patterns provide confidence that our samples were pure and reflect the true expression patterns of these tissues (see *Testis- and ovary-specific gene expression patterns in N. vitripennis* for further validation).

**Figure 2:**
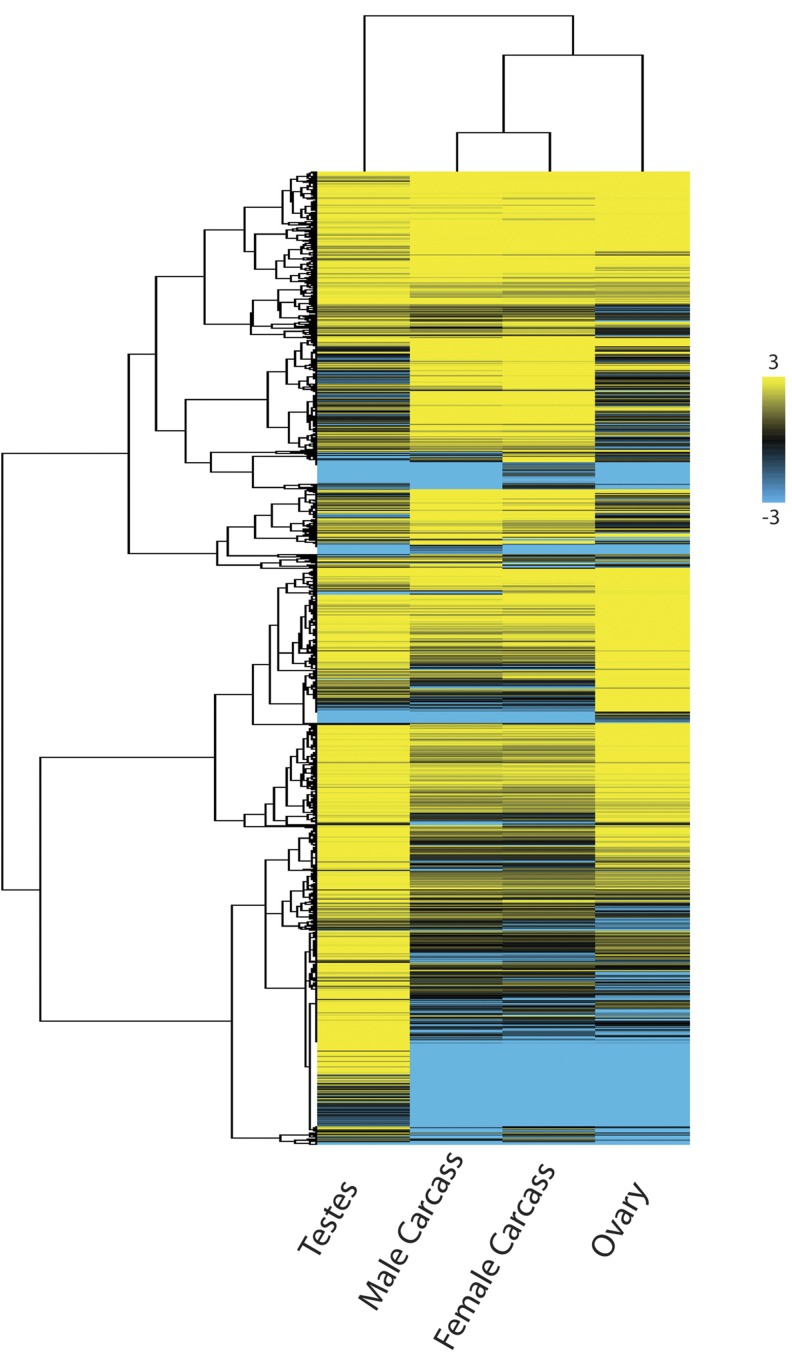
Heat map of expressed genes in *Nasonia* tissues. Clustering patterns show that gene expression in the male and female somatic tissues conditions are more similar to each other, while the ovary is more similar to the somatic conditions, and the testis is the outlier. The testis contains a large number of uniquely expressed genes compared to the other three tissue conditions (see bottom of map). Genes of similar expression patterns and levels are clustered together on the Y-axis (tree).

The expression profiles of the two somatic tissues are more similar to each other than to the germ-line tissues. The ovary is more similar to the somatic tissues, whereas the testis harbors the most distinct expression pattern (Figure 2). Specifically, of these four tissue conditions, the testis contains the largest number of uniquely expressed genes (Figure 2). The fact that the numbers of genes expressed in the germ-line tissues are similar to those in the somatic tissues underscores the complexity of genome-wide gene expression present in each germ-line tissue compared with the soma of each sex, which contain a combination of multiple differentiated somatic tissues. More specifically, we performed gene ontogeny analysis of our four tissue conditions specifically looking at genes that are expressed above 10 FPKM in each tissue type (Table S4, Table S5, Table S6, and Table S7). We discovered that the testis is enriched in 1427 Gene Ontology (GO) categories (Q value < 0.05) including genes involved in spermatogenesis (GO:0007283), spermatid cell differentiation (GO:0048515), and sperm individualization (GO:0007291) among others (Table S4), whereas the ovary is enriched in 1386 GO categories (Q value < 0.05) including genes involved in germarium-derived egg chamber formation (GO:0007293) and ovarian nurse cell to oocyte transport (GO:0007300) and germ-line cyst formation (GO:0048134) among others (Table S5). The somatic tissues of male adults without germ line contained enrichment of 860 GO categories (Q value < 0.05) including genes involved in immunity genes (GO:0002764, GO:0002757, GO:0002758, GO:0002218, GO:0045088), as well as cuticle genes (GO:0048079) among others (Table S6). The somatic tissues of the female carcass without germ line contained enrichment of 894 GO categories (Q value < 0.05) including genes involved in metabolism (GO:0008152, GO:0072521, GO:0032787, GO:0019538) and mitochondria energy production (GO:0005739, GO:0044429, GO:0044429, GO:0005759) among others (Table S7). These patterns corroborate findings from a recent study (Wang *et al.* 2015) and demonstrate that these previously observed gene expression patterns stem from the somatic tissues. We deposited all reads for each of the four tissues into an open access web browser for visualization of identified transcripts to their respective gene models (www.nasonia.caltech.edu).

### Testis- and ovary-specific gene expression patterns in *N. vitripennis*

Because the known cellular features unique to haplo-diploid development are restricted to the germ-line tissues, we further focused our analyses on the testis and ovary. Specifically, we sought to identify genes that are uniquely expressed in each of these tissues (Table S8 and Table S9), in addition to genes expressed in both germ-line tissues but not the somatic tissues (Table S10). Such genes may play specific roles in gametogenesis and haplo-diploid development. To uncover these genes, we subtracted away those expressed in the germ-line tissues that are also expressed in the soma. Of the remaining genes, we identified genes expressed uniquely in either testis or ovary; the remaining genes are those expressed in both germ-line tissues. Through this approach we uncovered 156 protein-coding genes that are exclusive to the testis (FPKM ≥1). In contrast, 22 protein-coding genes are expressed uniquely in the ovary and 40 genes are expressed in both germ-line tissues but not somatic tissues (Table S8, Table S9, and Table S10). The much higher number of testis-specific, protein-coding genes compared with the other two tissue conditions may be attributable to the complex architectures of the sperm tail and mitochondrial derivatives (Tokuyasu 1974; Turner 2003; Werner and Simmons 2008). In support of this idea, three testis-specific genes are predicted to be involved in axoneme structure of the sperm tail (NV16768, NV22584, NV21740), eight are mitochondria-related genes (NV23062, NV111110, NV16399, NV22869, NV13451, NV17359, NV10167, NV19180), and 19 belong to general metabolic processes (Table S8). One testis-specific gene, NV21361, is orthologous to the replication licensing factor MCM8 and may play a unique role the specialized mitotic divisions that produce sperm. Interestingly, in each of the three germ-line tissue conditions, the largest class of genes includes those of unknown orthology and predicted function; the testis contains the largest number of these genes compared to our other tissue conditions (Table S8).

We aspired to identify any chromatin-associated genes that are uniquely expressed in the testis because sperm cells are known to undergo multiple chromatin state changes preceding sperm maturation (Govin *et al.* 2004; Hennig and Weyrich 2013). One chromatin gene class of strong interest includes the protamines, which encode highly basic proteins that package DNA into an exceptionally condensed state in mature sperm (Eirin-Lopez *et al.* 2006; Balhorn 2007). However, these proteins are difficult to identify bioinformatically because of their high levels of sequence divergence among taxa (Rooney and Zhang 1999; Kasinsky *et al.* 2011). To identify protamine candidates, we first found the genes present in the *N. vitripennis* genome that contain an Arginine content of equal to or greater than 15%, which is the level present in the two *D. melanogaster* protamines, ProtA and ProtB (Jayaramaiah Raja and Renkawitz-Pohl 2005). We then screened these wasp genes for ones that are exclusively expressed in the testis (>10 FPKM in testes and <1 FPKM in any other tissue). Through this approach, we found eight such genes. The most abundant transcript that is uniquely present in the testis corresponds to a gene (NV31252; FPKM > 9,000) that was previously annotated in the Nvit_V2.1 genome as a protamine-like gene. The other seven (NV20435, NV23075, NV25929, NV50250, NV13264, NV23681, NV12597), all annotated as genes of unknown function, were expressed at lower levels. Additionally, we identified single gene variants of histone H2A (NV50148) and histone H2B (NV13549) as well as the linker histone H1 (NV13264), which are specifically expressed in the testis (Table S8). The testis-specific histone H2A and H2B transcripts were present at low levels; this pattern may reflect the fact that many core histone transcripts are not polyadenylated (Marzluff *et al.* 2008). Thus, these transcripts may actually be more abundant than our data reflect and are present due to carry-over from our selection of polyadenylated transcripts (see *Materials and Methods*). Nevertheless, these genes are testis-specific since their corresponding transcripts are not present in the ovary or somatic tissue conditions. In contrast, some histone variants such as H1.0 are known to be polyadenylated (Marzluff *et al.* 2008), and therefore, the transcript levels of the testis-specific histone H1 may reflect more biologically relevant levels. We also identified a testis-specific *N*-acetyltransferase (NV17675), which may be involved in post-translational modification of histones (Table S8).

Several genes unique to the ovary are a histone H1 variant (NV18954) and two transposable element-related genes (NV30938 and NV30926) (Table S9). However, the majority of uniquely expressed genes in the ovary are of unknown function. Both ovary and testis contained a histone H2B gene (NV10349) that was expressed uniquely in these tissues but not in the somatic tissue of either sex (Table S10). Additionally, a substantial number of transcripts were found only in the testis and ovary but do not map to previously annotated genes. Because these transcripts have poor protein coding potential they likely represent new transcribed sequences derived from noncoding euchromatic regions or, alternatively, from regions of heterochromatin that are rich in complex satellite DNA repeats and other repetitive sequences (Akbari *et al.* 2013) (Table S8, Table S9, and Table S10).

**Figure 3:**
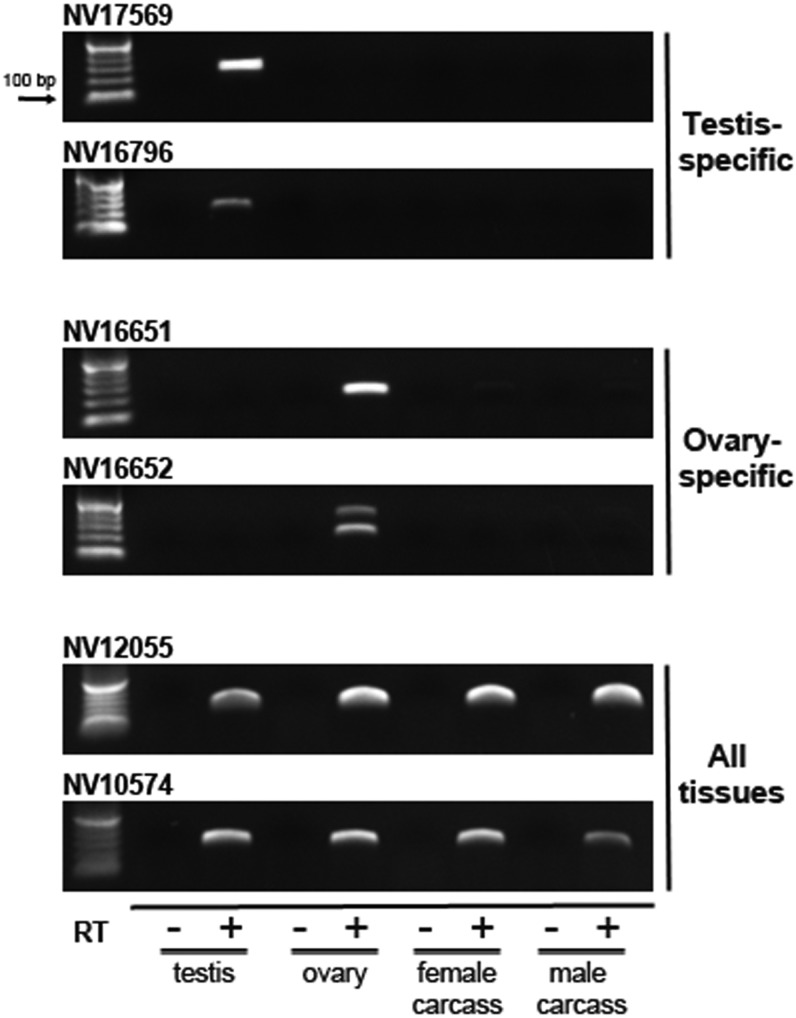
Reverse transcription-polymerase chain reaction (RT-PCR) validation of tissue-specific transcript patterns. RT-PCR was used to validate the expression patterns of a subset of genes from the RNA-Seq data sets. In all cases, the genes matched the patterns obtained from RNA-Seq. The amplified product of ovary-specific transcript from NV16651 appears as a doublet, which may result from alternative splicing. The arrow indicates the size of the lowest marker band.

To validate the tissue-specific expression patterns of our RNA-Seq data we performed RT-PCR on several testis- and ovary-specific transcripts as well as transcripts present in all four of the tissue conditions. In all cases the RT-PCR patterns matched those found with RNA-Seq (Figure 3). These results, in conjunction with the fact that several wasp genes match germ-line−specific expression patterns to those present in *D. melanogaster* (Graveley *et al.* 2011), help to demonstrate the robustness of our data and analyses (Table S8, Table S9, and Table S10).

### Investigating haplo-diploidy with transcriptomics

Haplo-diploid reproduction involves specialized variations of certain cellular processes in the germ-line tissues of hymenopteran insects. For example, sperm production in these insects involves a modified form of mitosis instead of meiosis, whereas oogenesis involves conventional meiosis (Whiting 1968). We hypothesized that large-scale expression differences of individual meiosis-related genes could in part underlie the specialized form of mitosis that occurs in lieu of meiosis in the wasp male germ line. In particular, certain meiosis genes may not be expressed in the wasp male germ line given that true meiosis does not occur in this sex. To begin to address this hypothesis, we determined the expression patterns of seventeen conserved meiosis-related genes in the *N. vitripennis* testis and ovary (Table S11); these genes are the previously predicted orthologs of meiosis-related genes in *D. melanogaster* (FLYBASE: FB2015_03). Of these seventeen wasp genes, eleven exhibited similar expression patterns compared to those found in *D. melanogaster* (data not shown). However, the remaining five genes showed different expression patterns in the wasp; four of these genes, including orthologs of mei-P26 (NV10527), mei-9 (NV15404), mamo (NV14750), and wisp (NV10736), which are expressed either preferentially or exclusively in the ovary of *D. melanogaster*, are present in relatively proportional amounts in both germ-line tissues, or primarily in the testis of *N. vitripennis* (Table S10). One gene, aust (NV12456), has enriched expression in the wasp germ-line tissues but solely in the ovary of *D. melanogaster*. Isoforms of subito (NV24472) and mamo (NV14749) are expressed primarily in the testis. We found no cases in which a gene was expressed in both germ-line tissues in *D. melanogaster* but expressed only in the wasp ovary (*i.e.*, not in the testis). These results reveal substantial expression differences in meiosis-related genes between wasp and fly, with a trend toward testis-specific expression in the wasp, although many of these genes show similar expression patterns between these organisms.

We also examined the expression patterns of genes known in other organisms to function in centrosome structure and dynamics as a way of assessing if *de novo* centrosome formation in the hymenopteran egg, a process important for development of unfertilized eggs into haploid males, may be governed by differential gene expression. Specifically, we identified the wasp orthologs of genes encoding core and peripherally associated centrosome factors in *D. melanogaster* (Alves-Cruzeiro *et al.* 2014) and examined their expression levels across our wasp transcriptome data sets. Of 348 known *D. melanogaster* centrosome-related genes, 238 have obvious counterparts in *N. vitripennis*, 25 of which are present in duplicated form (Table S12). All but 15 of these 238 genes show substantial expression levels in all four of our tissue conditions (Table S12). These 15 remaining wasp genes exhibit preferential or sole expression in both germ-line tissues or preferential expression in one of the germ-line tissues (Table S12). However, only three of these 15 genes show expression patterns that are notably different between *N. vitripennis* and *D. melanogaster*. For example, ana3 (NV11755) is expressed primarily in the ovary in *D. melanogaster* and in the testis in *N. vitripennis*. Two genes, cep135 (NV13344) and imp (NV17363), present in both germ-line tissues in flies, are expressed almost exclusively in the wasp testis. Finally, a copy of rpl23A (NV11027) is preferentially expressed in the fly testis while its wasp counterpart is expressed in all tissue conditions. These analyses demonstrate that a much smaller proportion of the examined centrosome-associated genes in *N. vitripennis* are differentially expressed between the germ-line tissues compared with meiosis-related genes in this organism.

## Discussion

During the past decade or more, a number of biological studies have been conducted in the jewel wasp *N. vitripennis*. Important genetic and genomic resources, including a high-quality genome sequence (Werren *et al.* 2010), have facilitated these and other such studies, thereby progressively making the wasp a preferred experimental system for hymenopteran-related biology. In this study we have produced the transcriptomes of the *N. vitripennis* ovary, female adult soma, and male adult soma to complement a previously produced transcriptome of the wasp testis (Akbari *et al.* 2013). These data, representing the comprehensive sets of genes expressed in each of these tissue conditions, have been made available as a research tool. Specifically, we have deposited these sequences into an open- access genome browser for straightforward viewing of transcripts relative to their gene models (www.nasonia.caltech.edu). Such transcriptome-based data sets will help to facilitate ongoing and new studies ranging from analysis of expression patterns of individual genes to broader comparisons of genome-wide gene expression patterns among *N. vitripennis* and other organisms.

Such transcriptome data sets can, in principle, corroborate genes predicted to exist in sequenced genomes but they also can confirm in which tissue(s) individual genes are expressed. For example, we have confirmed that NV31252, which was annotated as a protamine-like gene, (*i*) has one of the greatest arginine contents of any gene in the *N. vitripennis* genome, (*ii*) is uniquely expressed in the wasp testis, and (*iii*) exhibits the greatest expression level of those genes that are uniquely expressed in this tissue. Thus, our data strongly support the prediction that this gene is, indeed, a protamine. This conclusion strongly justifies further research efforts aimed at testing this hypothesis through transgenic and molecular experimental approaches. We also found seven additional genes that are uniquely expressed in the testis and also have unusually high arginine content; thus, these genes also are strong candidates for encoding protamines or other chromatin associated proteins, especially given that at least two protamines are encoded in the *D. melanogaster* genome (Jayaramaiah Raja and Renkawitz-Pohl 2005). Our studies also have uncovered a number of testis-specific histones that may play roles in the chromatin state changes that occur during spermatogenesis.

The gene expression patterns in our adult somatic tissue conditions generally reflect those recently reported in an RNA-Seq−based study of whole male and female adult wasps (Wang *et al.* 2015). Because our somatic tissue samples were without germ line, it is reasonable to conclude that the previously observed enrichment of immunity, venom, and pheromone signaling genes in whole adult males stems from expression in the soma and not the germ line. Additionally, it is likely that many of the testis-specific genes detected in our study were not previously detected in whole adult males (Wang *et al.* 2015) because the testes of adult males have completed the production of all sperm and therefore they do not contain the transcriptionally active periods of the dividing cystocytes. Broadly, transcriptome profiling of individual tissue conditions in this study as well as whole animals performed previously (Wang *et al.* 2015) provide complementary data sets that will allow for a more complete portrayal of gene expression patterns in *N. vitripennis*.

The testis- and ovary-specific transcriptomes can be further used for comparative functional studies across organisms. As an example, we explored whether two different processes tied to haplodiploidy—*de novo* centrosome formation in the female germ line and the production of sperm without meiosis in the male germ line—involve differences in expression of conserved genes between the testis and ovary. Specifically, we compared germ-line expression patterns of centrosome- and meiosis-related orthologs present in *N. vitripennis* and *D. melanogaster* to assess whether these genes show dramatically different expression patterns in the germ-line tissues of these organisms. Very few centrosome-associated genes show substantial differences in expression compared to *D. melanogaster*. In fact, only four genes—ana3, cep135, imp, and rpl23A—show dramatically distinct expression patterns in the wasp compared with those found in flies. All four of these genes show testis-biased expression in *N. vitripennis* whereas in *D. melanogaster* they are either exclusively expressed in ovary or in both germ-line tissues. It is possible that in *N. vitripennis* these genes may play important roles in the formation and function of the sperm tail’s axoneme, which is microtubule-based and originates from the centriole-derived basal body (Tokuyasu 1974; Schatten 1994; Turner 2003; Werner and Simmons 2008). This scenario would account for the absence of expression of these genes in the ovary. Interestingly, aside from these four centrosome-associated genes, all others examined were similarly expressed in both wasp germ-line tissues. These patterns intimate that the centrosome machinery is in large part the same between the two germ-line tissues, despite that the ovary specifically has the capacity to support *de novo* centrosome formation.

In contrast, five of 16 meiosis-related genes, which are expressed in the ovary or in both germ-line tissues of *D. melanogaster*, are testis-specific in *N. vitripennis*. Thus, a proportionally larger amount of genes show expression differences between these organisms compared with centrosome genes. We hypothesized originally that the lack of true meiosis in *N. vitripennis* males may have translated into more meiosis-related orthologs being expressed in the ovary compared with the testis. Our finding of the opposite pattern—that there are more testis-specific genes related to meiosis in *N. vitripennis*—argues that the mitotic production of wasp sperm does not result simply from the lack of expression of meiosis-related genes in the testis. Instead, the mitotic divisions giving rise to sperm in this organism may be more complicated than previously expected.

Our study contains a few important caveats worth consideration. First, our approach was aimed at examination of candidate genes whose functions are known from previous work in *D. melanogaster* and other model organisms. Therefore, other previously uncharacterized genes that may participate in haplo-diploid related processes at the transcriptional level, such as *de novo* centrosome formation, would not be picked up in our study. Second, our analyses focused primarily on gene expression differences that are between 100-fold and complete expression in one tissue or the other because of our experimental design. Therefore, our study does not include subtler expression differences that could in principle play roles in regulation of haplo-diploid processes between the germ-line tissues. Nevertheless, the data presented here allow for the identification of genes expressed solely in the germ-line tissues. Additionally, they provide some interesting patterns that can be pursued in subsequent studies, including more quantitative analyses of individual genes of interest, and they add to a growing number of resources in this bourgeoning model organism.

## Supplementary Material

Supporting Information
