# Supplementary material for: Identification of Genes Uniquely Expressed in the Germ-Line Tissues of the Jewel Wasp Nasonia vitripennis
Source: G3 (Bethesda). 2015 Oct 9;5(12):2647–53. doi: 10.1534/g3.115.021386 (PMC4683638; doi:10.1534/g3.115.021386)
Supplement: Supporting Information [file supp_5_12_2647__index.html]

Identification of Genes Uniquely Expressed in the Germ-Line Tissues of the Jewel Wasp Nasonia vitripennis — Supporting Information 

# Identification of Genes Uniquely Expressed in the Germ-Line Tissues of the Jewel Wasp *Nasonia vitripennis*

## Supporting Information for Ferree *et al.*, 2015

**Files in this Data Supplement:**

- Table S1 - Mapping Statistics. Mapping statistics for all samples indicating the total reads which map to junctions, exons, to multiple locations, uniquely and total locations mapped (.xlsx, 11 KB).
- Table S2 - Gene expression (.xlsx, 3,898 KB).
- Table S3 - Transcript expression (.xlsx, 3,163 KB).
- Table S4 - Gene Ontology and PFAM domain enrichment of all genes that are expressed > 10 FPKM in the testes with Q<0.05 (.xlsx, 504 KB).
- Table S5 - Gene Ontology and PFAM domain enrichment of all genes that are expressed > 10 FPKM in the ovary with Q<0.05 (.xlsx, 493 KB).
- Table S6 - Gene Ontology and PFAM domain enrichment of all genes that are expressed > 10 FPKM in the male carcass with Q<0.05 (.xlsx, 282 KB).
- Table S7 - Gene Ontology and PFAM domain enrichment of all genes that are expressed > 10 FPKM in the female carcass with Q<0.05 (.xlsx, 286 KB).
- Table S8 - Testes-exclusive transcripts. Requirements: Expression = 0 in all samples other than testes; RPKM ≥ 1 in testes. Total number of genes = 338 (.xlsx, 79 KB).
- Table S9 - Ovary-exclusive transcripts. Requirements: Expression = 0 in all samples other than ovary; RPKM ≥ 1 in ovary. Total number of genes = 22 (.xlsx, 68 KB).
- Table S10 - Germline-exclusive expression. Requirements: Expression = 0 expression in both the male/female somatic carcass samples; RPKM ≥ 1 in testes and ovaries. Total number of genes = 40 (.xlsx, 47 KB).
- Table S11 - Meiosis related gene expression (.xlsx, 65 KB).
- Table S12 - Centrosomal related gene expression (.xlsx, 100 KB).
